# Vaccine Acceptance and Hesitancy among College Students in Nevada: A State-Wide Cross-Sectional Study

**DOI:** 10.3390/vaccines10010105

**Published:** 2022-01-11

**Authors:** Leslie Elliott, Kanyeemengtiang Yang

**Affiliations:** School of Public Health, University of Nevada, Reno, NV 89557, USA; kyang@nevada.unr.edu

**Keywords:** COVID-19, vaccine hesitancy, vaccine acceptance, students

## Abstract

The purpose of this study was to identify factors related to COVID-19 vaccine acceptance and hesitancy in a diverse state-wide population of students. An electronic survey was emailed to students in the Nevada System of Higher Education to assess effects of the pandemic. The survey included questions related to vaccine status, interest in receiving the COVID-19 vaccine, factors influencing these decisions, and sources of health information. Among the 3773 respondents, over half (54%) were accepting of the vaccine, including vaccinated students (18.9%). Nearly one quarter (23.5%) expressed hesitancy to receive the vaccine, citing concerns about side effects and the need for more research. Factors related to hesitancy included female gender, increasing age, place of residence, marital status, and Black or Native American race. Vaccine hesitant respondents were less likely than other respondents to rely on public health agencies or newspapers for health information, and more likely to rely on employers, clinics, or “no one”. Culturally appropriate efforts involving COVID-19 vaccine information and distribution should target certain groups, focusing on factors such as side effects, development and testing of the vaccine. Research should investigate sources of health information of people who are hesitant to receive vaccines.

## 1. Introduction

In December 2020, the first vaccines for COVID-19 were approved for emergency use in the United States (U.S.) after initial phases of clinical trials suggested high efficacy [1,2]. Initial rollout of a vaccination plan was fraught with difficulties [3]; however, by late October 2021, approximately 190 million people (approximately 57.2% of the population) in the United States were fully vaccinated against the novel SARS-CoV2 virus that causes COVID-19, with 220 million (approximately 66.2% of the population) having received at least one dose of a vaccine [4]. In May 2021, a COVID-19 vaccine was authorized for use in adolescents 12 through 15 years of age [2]. Younger age groups have represented the lowest proportions of fully vaccinated groups, with increasing proportions in advancing age groups [4]. This trend reflects the initial prioritization of older populations in receiving the vaccine.

Now that vaccines are readily available to the U.S. population ages 12 years and older, questions about mandatory vaccination have arisen to address the increased incidence of COVID-19 in younger groups who may pose a risk for continued community transmission because of lower adherence to mitigation strategies [5,6]. Early pre-vaccine surveys suggested that younger populations are the most reluctant to receive a COVID-19 vaccine, with 43% of young adults (18 to 29 years) wanting to observe how it has worked for others before getting it themselves [7]. Vaccine hesitancy has also been identified in Black and Hispanic adults, urban residents, and Republicans [7]. However, most surveys were administered before the release of the COVID-19 vaccine.

To identify effects of the COVID-19 pandemic among young adults, a state-wide cross-sectional study was conducted among students enrolled in institutions of higher education in Nevada. In general, students enrolled in educational institutions comprise diverse populations across most characteristics, including race, age, socio-economic status, health, marital status, and political affiliation. This diversity is most obvious across Nevada’s state-wide educational system, which serves a broad population through doctoral-granting universities, community colleges, state colleges, and online programs that reach across state lines. The timing of this cross-sectional study afforded the opportunity to identify factors related to COVID-19 vaccine acceptance and hesitancy after people had been given the opportunity to observe distribution of the vaccine.

## 2. Materials and Methods

A survey with questions related to the effects of the COVID-19 pandemic on social determinants of health (economic stability, education, health and healthcare, and other factors often associated with health inequities) was distributed to students at six of the seven institutions in the Nevada System of Higher Education (“NSHE”), which comprises two doctoral-granting universities, a state college, and four community colleges. In addition to questions about social determinants of health, the survey also included questions related to COVID-19 test status, vaccine status, and interest in receiving the COVID-19 vaccine. All institutions in the NSHE system were invited to participate in the survey, and only one of the seven institutions declined because they did not want to over-burden the students.

Contact persons (e.g., administrative or research staff) at each of the participating institutions decided whether to distribute the email survey from their own institution or to share the student email distribution list with the researchers. Regardless of the method of distribution, recruitment emails with a link to the study survey were distributed in February and March, 2021, to all students with active emails at the following institutions: College of Southern Nevada (“CSN”, *n* = 25,544), Great Basin College (“GBC”, *n* = 2199), Nevada State College (“NSC”, *n* = 4017), University of Nevada, Las Vegas (“UNLV”, *n* = 43,827), University of Nevada, Reno (“UNR”, *n* = 18,450), and Western Nevada College (“WNC”, *n* = 2265). Emails were distributed either by the investigators through the UNR Qualtrics (Qualtrics, Provo, UT) email system [8], using email lists provided by the institution (GBC, UNR undergraduates, CSN, WNC), or by administrative staff at their respective institutions (UNR graduates, NSC, UNLV). Emails sent through UNR Qualtrics included reminders each week for approximately three weeks, since the Qualtrics system had the capacity to send reminders to non-responding email recipients.

Students were eligible to participate if they were 18 years or older. The study was determined by the UNR Institutional Review Board on 16 November 2020 (1679042-1) as exempt from IRB review.

### Statistical Analysis

Data were downloaded from the Qualtrics survey system and analyzed using Stata/SE 16.1 for Windows [9]. Vaccine hesitancy was defined as having answered “somewhat reluctant” or “very reluctant” on a question about eagerness to receive the COVID-19 vaccine, compared with “somewhat eager”, “very eager”, “indifferent”, and having received the vaccine already. Vaccine acceptance was defined as having received the vaccine or having answered “somewhat eager” or “very eager” to receive the vaccine. Indicator variables were created for all categorical variables (age group, race, student status, residential status, race, and marital status), and logistic regression was used to evaluate relationships between vaccine hesitancy and these factors.

## 3. Results

The initial response proportion for all institutions combined was 4.07% (3900/95,870), accounting for duplicate (*n* = 83) and bounced (*n* = 267) emails reported in the Qualtrics system. After responses of students <18 years of age (*n* = 26) and incomplete surveys (*n* = 101) were removed from the dataset, 3773 responses remained for analysis. This represented approximately 3.9% of the students receiving recruitment emails (*n* = 95,870). The institution-specific responses ranged from 2.0% (NSC) to 7.7% (GBC). The majority of respondents were undergraduates (82.3%), 30 years and younger (63.6%), female (67.5%), and never married (61.9%) (Table 1). More than half (54.2%) reported living in a large city.

Of all respondents, 16.5% (*n* = 624) reported having tested positive for COVID-19, which differed by race, residential status, and student status, but not by age, gender, or marital status (Table 2). Student groups that reported lower percentages of positive tests included Asians, Native Hawaiian/Pacific Islanders, those living in rural or suburban areas, and those in doctoral or postdoctoral programs. Of respondents who had not been tested for COVID-19, another 16% (*n* = 502) suspected that they had contracted COVID-19 but were not tested. This was similar for all groups except for Asians, who were less likely than Whites (OR 0.59, 95% CI 0.40 to 0.85; *p* = 0.005) to have reported this.

At the time of the survey, 714 (18.9%) of the student respondents had received a COVID-19 vaccine. Although vaccination status did not differ by race or residence, there were substantive differences by age, student status, and marital status (data not shown). Positive vaccination status increased with each decade of age, with students >50 years of age 168% more likely to be vaccinated than students 18 to 25 years of age (OR 2.68, 95% CI 1.85 to 3.86). Undergraduate students were less likely to be vaccinated than master’s, doctoral, and postdoctoral students. Married students, or those living with a partner, were almost twice as likely as never married students to be vaccinated.

### Vaccine Hesitancy and Acceptance

Nearly one quarter of the respondents (23.5%) were hesitant to receive the vaccine. Of these, (*n* = 888), 40% were “somewhat reluctant” and 60% were “very reluctant”. Over half (54%) of the respondents were accepting of the vaccine, including those who had received the vaccine already (*n* = 714, 18.9%).

Vaccine hesitancy was prominent in specific racial groups, with American Indians and Blacks more than twice as hesitant as Whites to receive the vaccine, and Asians nearly half as hesitant (Table 3). Hesitancy increased with age, with students between 46 and 50 years reporting the most hesitancy compared with younger students (OR 2.95, 95% CI 1.77 to 4.92; *p* < 0.000). Students in rural areas and small towns were more hesitant than those in large cities. Undergraduate students were more hesitant to receive the vaccine than graduate students, which seemed to contradict the association between hesitancy and increasing age. We assessed vaccine hesitancy by age within categories of student status, and found strong associations between hesitancy and increasing age in the undergraduate group, which represented the largest subgroup. The undergraduates were more heterogeneous with respect to age than graduate students, accounting for the strong associations seen between hesitancy and increasing age in the entire study population. Females were more hesitant than males, and students who were living with a partner, married, widowed, divorced, or separated were more hesitant than students who were never married.

Vaccine hesitancy was not affected by whether the student or a family member had been hospitalized for COVID-19 (data not shown). Students who were reluctant to be vaccinated expressed concerns about vaccine side effects (67.3%), the need for more COVID-19 vaccine research (74.3%), and being suspicious of vaccines in general (25%) (Table 4). At the same time, these students reported a desire to protect themselves (15.4%), their friends and family members (16.8%), and to “help get things back to normal” (16.2%).

The more popular sources for obtaining information about health or COVID-19 were the CDC or other public health agency (34.3%) and social media (20.7%), followed by television and family or friends (Table 5). Compared with non-hesitant respondents, vaccine hesitant respondents were less likely to rely on public health agencies and the newspaper for health information and were more likely to rely on employers and clinics or doctors’ offices (Table 6). Vaccine hesitant respondents were over four times likely to report that they relied on “no one” and nearly three times more likely to rely on “other” sources.

## 4. Discussion

This statewide cross-sectional study of students throughout Nevada identified that approximately 21% of respondents had received a COVID-19 vaccine, resulting in an overall acceptance proportion (vaccinated plus eager to be vaccinated) of approximately 54%. Nearly 25% of unvaccinated individuals were reluctant to receive a COVID-19 vaccine. The most common reasons for reluctance to get the vaccine were related to the belief that more research was needed (74.3%) and concern about side effects (67.3%). Interestingly, among students who were reluctant to get the vaccine, approximately 15–17% said they wanted to protect themselves and family members; it is unclear whether these respondents meant they believed the vaccine to be harmful to self and others, or whether they were conflicted about their hesitancy and these reasons would eventually affect their decisions to receive the vaccine.

In reporting their most reliable source of health information, including COVID-19, vaccine hesitant and non-hesitant respondents differed in their preferences. Vaccine hesitant respondents were more likely to depend on clinics and doctors’ offices, employers, “no one”, and other sources, while vaccine accepting respondents were more likely to depend on the CDC or other public health agencies and newspapers. Interestingly, vaccine hesitant and accepting respondents did not differ substantially in their reliance on social media, television, and family and friends. A study among Egyptian healthcare workers found similar results regarding social media and television, and found that “willing” respondents were more likely than hesitant or refusing respondents to depend on international organizations websites (WHO, CDC) [10]. Vaccine hesitant respondents in a Norwegian population were more likely to favor unmonitored media platforms compared to source-verified platforms [11]. Similar to our results, negative vaccine intent among older Americans (Medicare enrollees) was associated with more reliance on healthcare providers; however, that study found an association between negative vaccine intent and social media, other internet pages, and family/friends [12].

Most of the surveys about vaccine acceptance and hesitancy were distributed before vaccines were approved and therefore reflect intent to get a vaccine once it became available. A systematic review of 126 studies published between January and October of 2020, found regional acceptance in the U.S. ranging from 38% in the northeast to 49% in the west [13]. The authors noted that, of the many demographic variables across the studies, a college education seemed to create the largest differences in vaccine intention (42% for those without a college education, 62% for those with a college education, and 75% among postgraduates). These estimates were similar to our findings in unvaccinated students; undergraduates were less eager (48%) than master’s (66.7%) and doctoral/postdoctoral students (77.2%) to receive the vaccine. Although overall vaccine acceptance was higher among students in our study, there were similarities for age, sex, residence, and race-ethnicity groups.

Few surveys have been conducted among college students of all ages, focusing mostly on specific groups, such as undergraduates or students in healthcare programs. In general, surveys among students have suggested high acceptance rates. For example, 647 undergraduates in the northwest United States reported in November 2020 greater intentions of getting a COVID-19 vaccine, once available, than an influenza vaccine (91.6% vs. 76.0%) [6]. A study of 457 students in New Jersey reported that 23% had received the vaccine while another 53% reported intention to receive the vaccine [14]. Among 237 students in Rhode Island, 92% were somewhat or very likely to receive the COVID-19 vaccine once eligible [15].

Vaccine acceptability surveys among students have been more prevalent in other countries, where acceptance is variable. For example, in a study of students in Saudi Arabia (*n* = 407), 49% had either received or were registered to receive the vaccine, and 90% of unvaccinated and unregistered students were eager to receive the vaccine [16]. This level of acceptance was similar to that in students in central and southern Italy (91.9%, 94.7%) [17,18]. Other studies have found somewhat lower levels of acceptance, such as 86% in Italian students [19], 83.4% in Vietnamese students [20], 78% in Canadian students [21], 76% in Chinese students [22], 69.3% in Ethiopian students [23], and 58% in French students [24]. Some of the variability might be explained by differences in student populations. For example, the age ranges were quite different across studies, and at least one study excluded students ages 25 years and older [24]. Variability in these populations may also be related to the timing of the studies in relation to the progression of COVID-19 in these countries, such as high acceptability among Italian students since Italy was the first western country to be heavily affected by the pandemic.

Reasons for vaccine acceptance and hesitancy in this study were similar to those in other studies. Students wanted to be vaccinated to protect themselves and others [15,16,20,21,24] and to resume a normal life [20,24], while hesitancy was related to concern about side effects [6,15,17,20,21,23,24,25] and expedited vaccine trials [6,17,21,24] or incomplete information [20,23]. Although our study did not ask about trust in government, high confidence in government and ongoing awareness efforts were reasons cited for a high level of acceptance in Saudi Arabian students [16], while recommendation by government was identified as a reason for vaccine refusal and hesitancy in American students [25]. A “rapid national assessment” of vaccine hesitancy in the United States (*n* = 1878) had similar findings to this study, such as higher hesitancy in African Americans, rural residents, females, and individuals with lower education [26]. That study found a strong association between political party and vaccine hesitancy, with Republicans, Independents and “Other” parties much more hesitant than Democrats.

To our knowledge, this is the largest study of U.S. students regarding COVID-19 effects and vaccination status and hesitancy. Despite the large sample size, the response proportion among all Nevada students was low (3.9%). The actual response proportion is difficult to evaluate for several reasons. Administrators shared email lists of students who were likely enrolled in the fall semester and had not matriculated in the spring semester; for example, comparisons of the number of emails sent with the final enrollment numbers from the institutions are different. Remarkably, the survey was sent to 43,827 students at UNLV, while the official enrollment numbers for spring 2021 show 28,395 students. Using the actual enrollment would increase the response at UNLV from 2.3% to 3.5%. Because UNLV had the largest population of students, the low response weighted heavily in the overall response proportion. Email lists included some students who were not eligible for the study (i.e., less than 18 years of age), and we did not have information about these numbers. While the survey was sent to 96,302 emails, the final enrollment for spring 2021 was 89,945 students, resulting in a response proportion of 4.2% (including the responses of students who were less than 18 years of age). This represents the lowest response proportion estimable but may still be an underestimate. Although low response can be an indicator of potential non-response bias, it is unlikely that the low response affected the outcomes of this specific study on vaccine hesitancy and acceptance since the recruitment email only mentioned the desire to obtain information on how the pandemic had affected the students’ health and wellbeing.

The investigators received numerous responses from the recruitment emails complaining about COVID in general and stating displeasure at receiving a survey about it, reflecting the overall fatigue most universities are observing among their students. During the previous fall semester, institutions sent numerous surveys to students about their experiences with online education after the mode of delivery had pivoted for all on-campus students. These surveys also had low response proportions, and we hoped that sending the survey early in the spring semester would yield a higher response since it was separated from the previous surveys. Because all surveys to the students had low responses, it is unlikely that the results of this survey reflect non-response bias related to the outcomes being measured. Despite the apparent low response proportion, however, the large sample size allowed meaningful comparisons among groups.

Compared with fall 2020 enrollment in all institutions of higher education in Nevada (“NSHE”) [27], the demographics in our study differed with respect to gender and most race-ethnicity groups. Assigning race without regard to ethnicity (to match NSHE reporting), our study enrolled more females (67.5% vs. 59%), more Whites (45.2% vs. 38%), and fewer Blacks (4.6% vs. 6.9%), individuals of multiple race (6.3% vs. 7.6%), and Hispanics (25.9% vs. 30%). Asian students and American Indian, Alaska Native students were similar across NSHE and our study populations. However, the differences between our study demographics and those of all NSHE institutions combined were likely affected by the different response proportions across the NSHE institutions, since the demographics differ across institutions. For example, 90% of the responses were from the College of Southern Nevada (CSN, 27%), University of Nevada, Reno (UNR, 37%) and University of Nevada, Las Vegas (UNLV, 26%), which show enrollments for Blacks as 10% vs. 3.3% vs. 8.0%, respectively. Demographics for spring 2021 enrollments have not been published yet, so it is not possible to evaluate whether demographics changed from fall 2020 to spring 2021.

This study was conducted in February and March of 2021, after the COVID-19 vaccine was available to most adults. Subsequently, campaigns have been conducted across the United States in efforts to convince and incentivize individuals to receive the COVID-19 vaccine. Where incentivization has not worked, employers, government agencies, and school systems have begun to mandate vaccines for employees, teachers, and students. While these mandates have convinced some individuals to receive the vaccine, they have mostly been met with widespread resistance. By fall 2021, thousands of workers had lost their jobs for not receiving the vaccine when mandated by their employer [28]. The NSHE system has adopted a policy to mandate vaccines for all students enrolling in classes for spring 2022.

## 5. Conclusions

This study represents a diverse population of students across the state of Nevada and has identified specific sub-groups and factors related to hesitancy. The findings suggest that efforts involving vaccine information and distribution should focus on certain groups, such as females, individuals living in rural areas and small towns, specific age groups, and individuals who are married/living with a partner, separated, or divorced. Campaigns that target these groups will need to focus on factors related to hesitancy, such as fear of side effects, lack of information about the vaccine, and protecting self and others. Culturally appropriate materials need to be developed for race-ethnicity groups identified to be most hesitant, such as Blacks and American Indians. Additional research should investigate sources of health information and how they affect vaccine hesitancy and acceptance. As vaccine mandates grow, further research should focus on understanding how individuals process risk–benefit decisions that factor into their decisions to receive a vaccine. This continues to be an important issue in public health as the COVID-19 pandemic continues to wax and wane with new variants of the virus. Understanding these factors may provide valuable information for dealing with future public health epidemics, as well as addressing the continuing perpetuation of the COVID-19 pandemic.

## Figures and Tables

**Table 1:** Characteristics of respondents in the NSHE student survey, February–March, 2021.

Characteristics	Number (%)
Age range, years	
18–20	1202 (31.6)
21–25	1108 (19.2)
26–30	486 (12.8)
31–35	314 (8.3)
36–40	241 (6.3)
41–45	159 (4.2)
46–50	98 (2.6)
51–55	71 (1.9)
>55	76 (2.0)
Missing	18 (0.5)
Gender	
Female	2546 (67.5)
Male	1012 (26.8)
Other	66 (1.8)
Missing, prefer not to answer	149 (4.0)
Race	
American Indian, Alaska Native	48 (1.27)
Asian	381 (10.1)
Black or African American	185 (4.9)
Multiple race	519 (13.8)
Native Hawaiian, Pacific Islander	33 (0.87)
White	2072 (54.9)
Missing, prefer not to answer	535 (14.2)
Ethnicity	
Hispanic or Latino or Spanish	973 (25.8)
Not Hisp. Or Latino or Spanish	2432 (64.5)
Missing, prefer not to answer	368 (9.8)
Marital status	
Living with partner	349 (9.3)
Married	680 (18.0)
Never married	2336 (61.9)
Separated/Divorced	183 (4.6)
Widowed	18 (0.5)
Missing, prefer not to answer	207 (5.5)
Residential environment	
Large city	2046 (54.2)
Rural area	201 (5.3)
Small town	511 (13.5)
Suburb area	960 (25.4)
Missing	55 (1.5)
Student status, spring 2021	
Undergraduate	3106 (82.3)
Master’s student	242 (6.4)
Doctoral, post-doctoral student	178 (4.8)
Not enrolled, taking courses	89 (2.4)
Missing	158 (4.2)

**Table 2:** COVID positive test results of respondents in the NSHE survey, February–March, 2021.

Characteristics	Number (%)	Odds Ratio (95% CI)	*p*-Value
Total Population	624 (16.5)		
Age (years)			
18–25	401 (17.4)	Referent	
26–35	131 (16.4)	0.92 (0.74–1.14)	0.44
36–45	61 (15.3)	0.83 (0.62–1.12)	0.23
46–50	13 (13.3)	0.69 (0.38–1.25)	0.22
>50	18 (12.2)	0.61 (0.37–1.01)	0.06
Gender			
Female	164 (16.2)	1.07 (0.88–1.31)	0.49
Male	440 (17.3)	Referent	
Other	14 (21.2)	1.33 (0.72–2.46)	0.37
Race			
American Indian, Alaska Native	11 (22.9)	0.80 (0.31–2.10)	0.65
Asian	42 (11.0)	0.59 (0.40–0.85)	0.005
Black or African American	31 (16.8)	0.98 (0.62–1.55)	0.93
Multiple race	84 (16.2)	1.12 (0.85–1.48)	0.42
Native Hawaiian, Pacific Islander	7 (21.2)	0.54 (0.12–2.37)	0.42
White	343 (16.6)	Referent	
Missing, prefer not to answer	106 (19.8)		
Marital Status			
Never married	408 (17.5)	Referent	
Married/Lives with Partner	169 (16.4)	0.90 (0.74–1.10)	0.31
Divorced/Separated/Widowed	15 (7.3)	0.86 (0.58–1.27)	0.45
Residential environment			
Large city	372 (18.2)	Referent	
Rural area	20 (9.9)	0.46 (0.29–0.76)	0.002
Small town	92 (18.0)	1.00 (0.78–1.29)	0.98
Suburb area	140 (14.6)	0.76 (0.62–0.95)	0.014
Student Status			
Undergraduate	536 (17.3)	Referent	
Master’s	38 (15.7)	0.84 (0.59–1.21)	0.35
Doctoral/Postdoctoral	18 (11.3)	0.58 (0.35–0.96)	0.04
Not enrolled in a program	32 (18.1)	1.07 (0.72–1.59)	0.75

**Table 3:** Vaccine hesitancy among students in Nevada, February–March, 2021.

Student Groups	Odds Ratio (95% Confidence Interval)	*p*-Value
Age range, years		
18–25	Referent	
26–35	1.57 (1.29–1.92)	<0.000
36–45	1.65 (1.26–2.15)	<0.000
46–50	2.95 (1.77–4.92)	<0.000
> 50	1.18 (0.71–1.76)	0.627
Race		
Whites	Referent	
American Indians	2.92 (1.48–5.76)	0.002
Asian	0.58 (0.42–0.80)	0.001
Black or African American	2.16 (1.52–3.08)	<0.000
Native Hawaiian, Pacific Islander	2.02 (0.92–4.47)	0.081
Multiple races	1.08 (0.86–1.37)	0.493
Residential area		
Large city	Referent	
Rural area	1.94 (1.38–2.73)	<0.000
Small town	1.36 (1.07–1.72)	0.012
Suburb area	0.79 (0.65–0.97)	0.022
Student status		
Undergraduate	Referent	
Master’s	0.64 (0.44–0.94)	0.021
Doctoral, Postdoctoral	0.28 (0.14–0.55)	<0.000
Not enrolled in program	1.26 (0.85–1.86)	0.247
Gender		
Male	Referent	
Female	1.27 (1.06–1.53)	0.009
Other	0.42 (0.27–1.12)	0.102
Marital status		
Never married	Referent	
Living with partner, married	1.52 (1.27–1.84)	<0.000
Widowed, divorced, separated	1.91 (1.35–2.8)	<0.000

**Table 4:** Reasons for eagerness or reluctance to vaccinate among students in Nevada, February–March, 2021.

	Reluctant Number (%)	Eager Number (%)	Indifferent Number (%)
Concerned about side-effects	598 (67.3)	272 (20.6)	190 (39.8)
Vaccine needs more research	660 (74.3)	157 (11.9)	226 (47.3)
Want to protect myself	137 (15.4)	1120 (84.7)	146 (30.5)
Want to protect friends and family members	149 (16.8)	1173 (88.7)	222 (46.4)
Suspicious of vaccines in general	222 (25.0)	28 (2.1)	51 (10.7)
Want things back to normal	144 (16.2)	1044 (78.9)	216 (45.2)

**Table 5:** Sources of information about health or COVID-19.

	Hesitant (*n* = 888)	Accepting (*n* = 2037)	Indifferent (*n* = 478)	Total (*n* = 3773)
CDC or other public health agency	273 (30.7)	880 (43.2)	139 (29.1)	1295 (34.3)
Social Media	186 (21.0)	459 (22.5)	130 (27.2)	781 (20.7)
Television	80 (9.0)	199 (9.8)	62 (13.0)	342 (9.1)
Family, significant other, friends	91 (10.3)	166 (8.2)	57 (11.9)	314 (8.3)
Other	85 (9.6)	70 (3.4)	26 (5.4)	183 (4.9)
Employer	57 (6.4)	99 (4.9)	11 (2.3)	168 (4.5)
Newspaper	14 (1.6)	90 (4.4)	14 (2.9)	118 (3.1)
Clinic, hospital, or doctor’s office	40 (4.5)	50 (2.5)	20 (4.2)	110 (2.9)
No One	60 (6.8)	24 (1.2)	16 (3.4)	103 (2.7)

**Table 6:** Associations between sources of health information and vaccine hesitancy.

	Odds Ratio (95% Confidence Interval) (Referent = Non-Hesitant)	*p*-Value
CDC or other public health agency	0.65 (0.55–0.77)	0
Social Media	0.87 (0.72–1.05)	0.135
Television	0.86 (0.66–1.11)	0.247
Family, significant other, friends	1.17 (0.91–1.52)	0.219
Other	2.67 (1.97–3.61)	0
Employer	1.50 (1.08–2.09)	0.016
Newspaper	0.37 (0.21–0.65)	0.001
Clinic, hospital, or doctor’s office	1.65 (1.11–2.45)	0.014
No one	4.49 (2.99–6.75)	0

## Data Availability

Data for this study may be requested by contacting the corresponding author.
